# Supplementary material for: Sizing nanomaterials in bio-fluids by cFRAP enables protein aggregation measurements and diagnosis of bio-barrier permeability
Source: Nat Commun. 2016 Sep 22;7:12982. doi: 10.1038/ncomms12982 (PMC5036146; doi:10.1038/ncomms12982)
Supplement: Supplementary Information — Supplementary Figures 1-14, Supplementary Table 1 and Supplementary Notes 1-5 [file ncomms12982-s1.pdf]

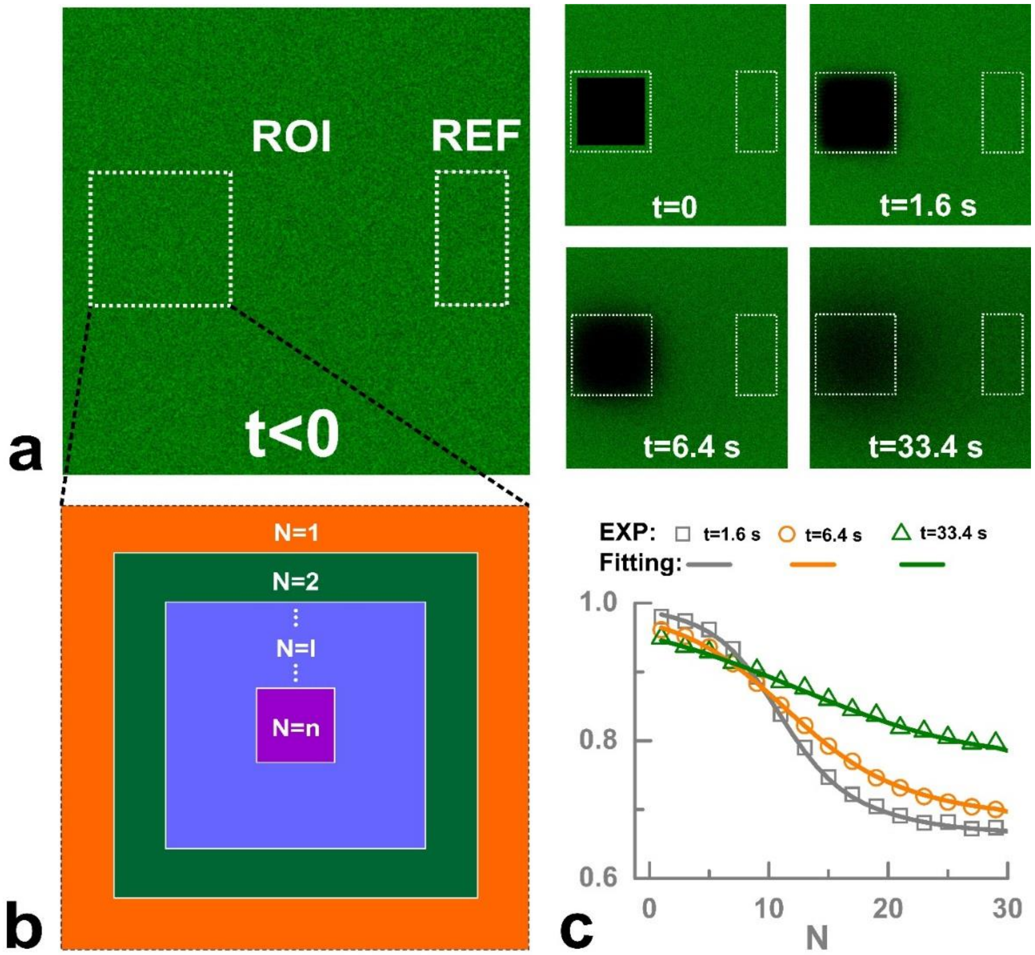

Supplementary Figure 1. (**a**) Confocal images (field of view 153.6 μm by 153.6 μm) are shown of an cFRAP experiment in a FITC-dextran solution. Images are shown before ($t < 0$), during ($t = 0$) and after photobleaching ($t > 0$). cFRAP analysis is performed on the indicated Region of Interest (ROI). The REF region indicates the reference area that is used in the analysis to correct for potential laser fluctuations and bleaching during imaging. (**b**) For analysis according to the 'ring-based method', the ROI is divided into *n* equally spaced rectangular ring areas. (**c**) The normalized average intensity of each 'ring' is shown at time points t = 1.6, 6.4 and 33.4 s. The solid lines represent the best fit of the cFRAP model. Note that a single fit is done to all spatial profiles for all available time points in the data set simultaneously.

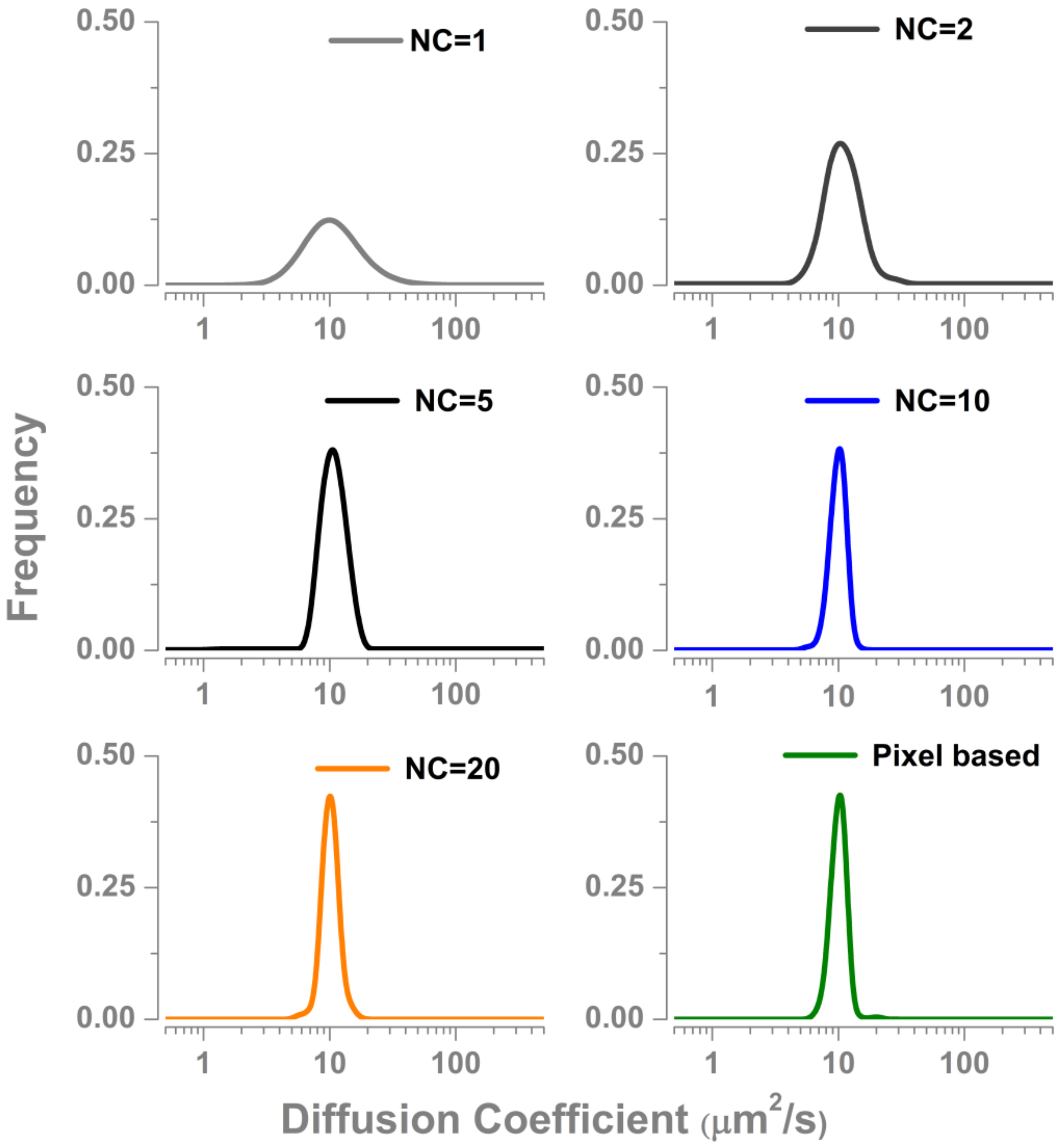

Supplementary Figure 2. Recovery images were simulated according to Eq (2) for a single component system of D = 10 μm^2 s^{-1}. D-distributions are shown for the ring-based method for different numbers of equally spaced rings (NC=1, 2, 5, 10 and 20) and the pixel-based method.

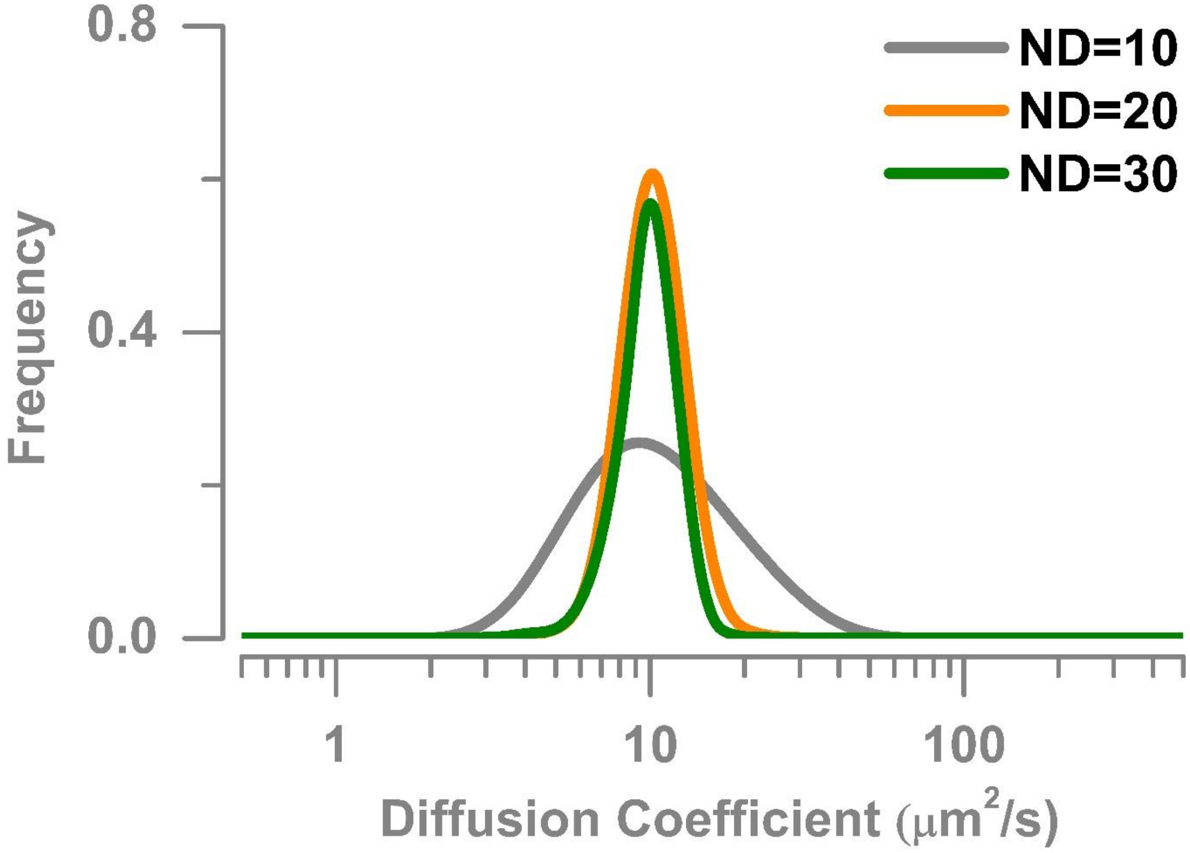

Supplementary Figure 3. The influence of discretization of D-space is evaluated for a single component *D*=10 $\mu m^2 s^{-1}$. *ND* is the number of D values that are chosen over three orders of magnitude with logarithmic spacing. *ND* was varied from 10 to 30.

.

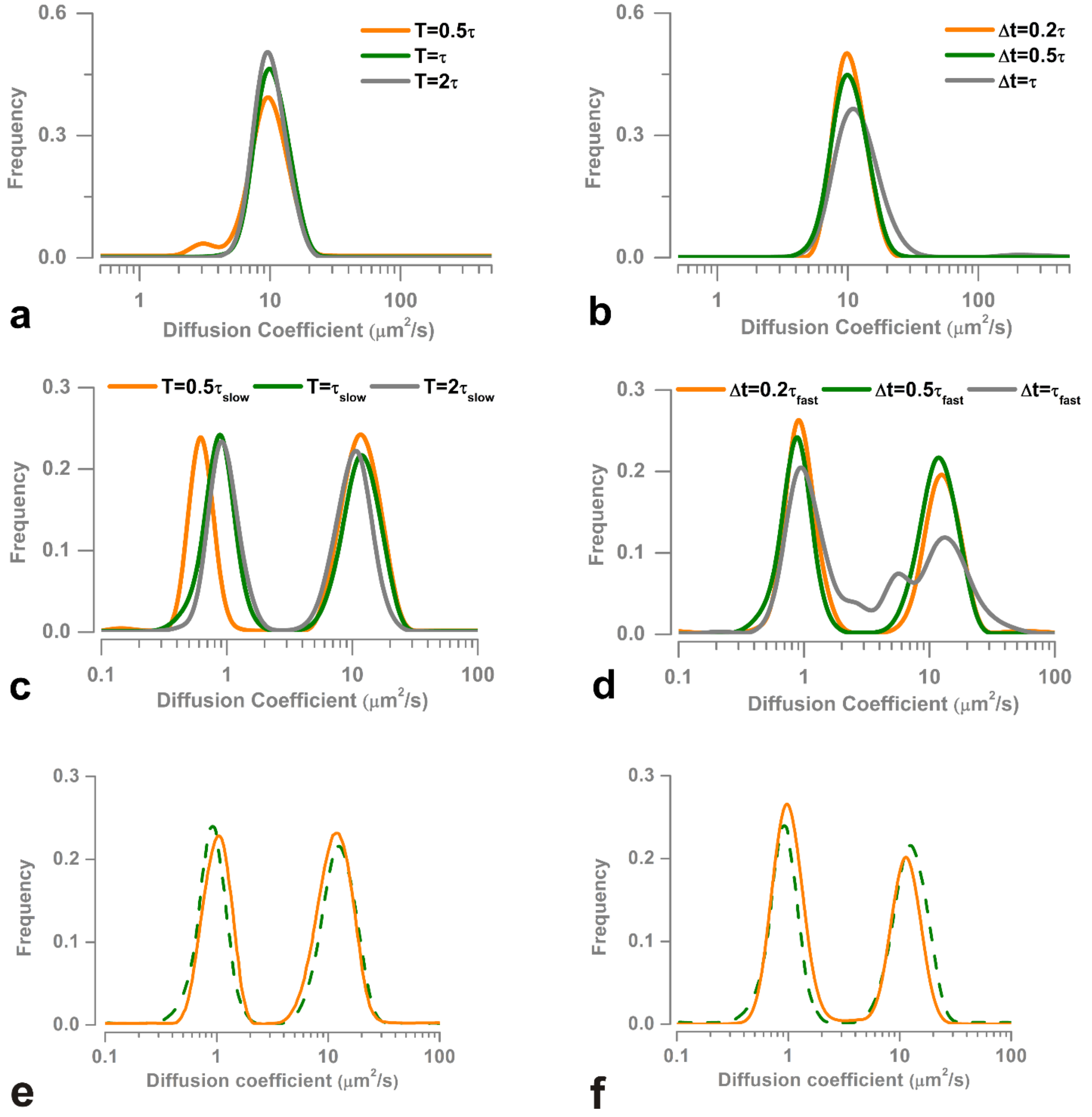

Supplementary Figure 4. The influence of total measurement time (T) and time interval (Δt) is evaluated for (**a, b**) a single component (D=10 μm^2 s^{-1}) and (**c, d**) a two-component system (D_1=1.0 μm^2 s^{-1}and D_2=10 μm^2 s^{-1}). (**e**) To limit the number of recovery images, the time interval Δt can be increased with ~20% per recovery image or (**f**) with 100% per every 5 frames. For ease of comparison, the dashed lines in **e** and **f** show the distributions calculated for T =τ_{slow}, Δt =0.5 τ_{fast} from **c** and **d**.

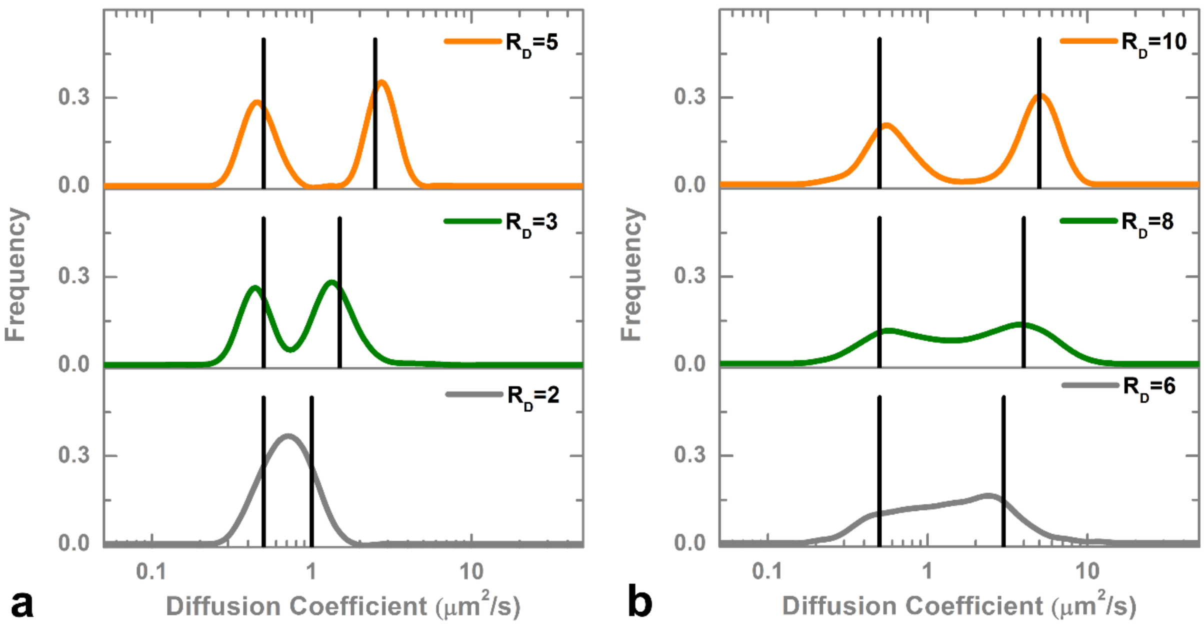

Supplementary Figure 5. The ability of cFRAP to discriminate two components was investigated using simulated recovery images. Different ratios of diffusion coefficients were evaluated using (**a**) the full tempo-spatial information vs. (**b**) using only time information as in standard FRAP analysis. R_D indicates the ratio of the two diffusion coefficients, where D_1=0.5 μm^2 s^{-1}. The black lines indicate the diffusion coefficients used in the simulations.

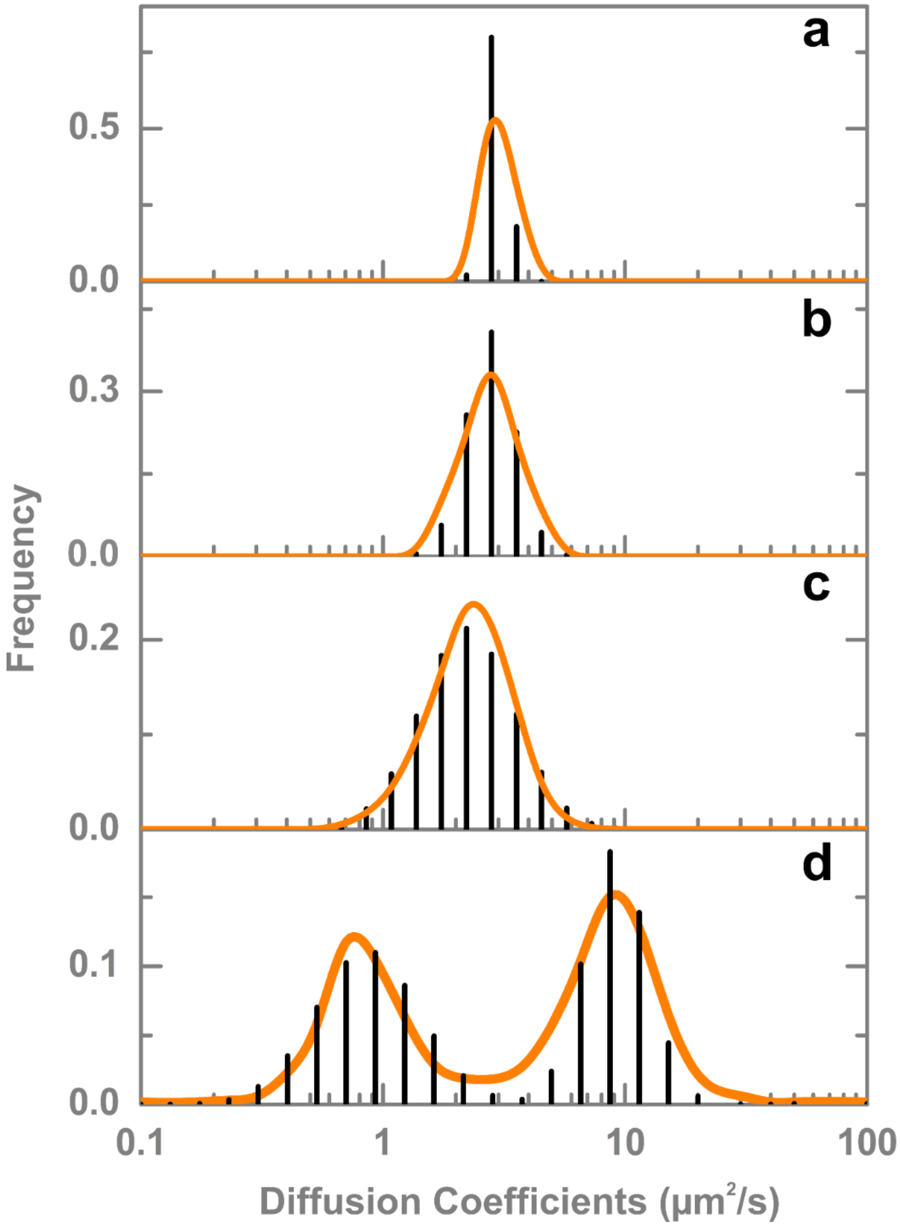

Supplementary Figure 6. The ability of cFRAP analysis to recover continuous *D* distribution was tested for two simulated polydisperse systems. As indicated by the black bars, (**a-c**) the first system follows a lognormal distribution with location parameter μ = 1.4 μm^2 s^{-1}and scale parameter σ varying from 0.1 (**a**), 0.5 (**b**) to 2.0 (**c**)and (**d**) the second follows a double lognormal distribution with μ_1=0.9 μm^2 s^{-1}, σ_1 =1.5 and μ_2=8.7 μm^2 s^{-1} σ_2=1.5.The orange curves are the result from cFRAP analysis and almost perfectly match with the true distributions.

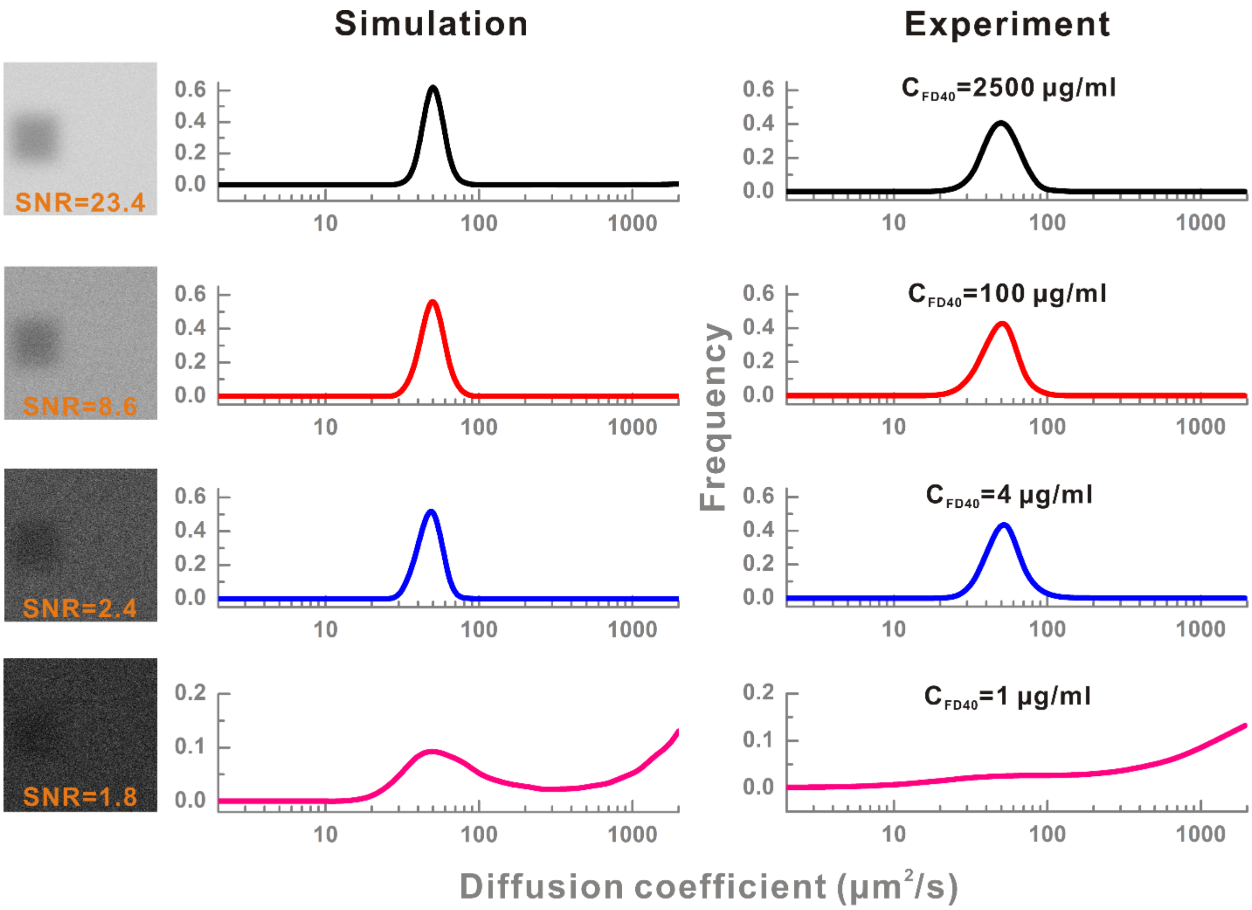

Supplementary Figure 7. The influence of signal to noise ratio (SNR) on cFRAP analysis and determination of the lower concentration limit. cFRAP was performed on solutions with decreasing FD40 concentration and, therefore, deteriorating SNR. Experimental results are cross-checked with simulated experiments (single component system with D=50 μm^2 s^{-1}) having similar SNR. Even at a SNR as low as 2.4, the distribution of diffusion coefficients could be retrieved by cFRAP. On the confocal microscope used in this study, this corresponded to a lower concentration of 4 μg ml^{-1} FD40 (100 nM). It is to be noted that the exact value of the lower concentration limit depends on the type of confocal microscope used and the brightness of the labelled molecules.

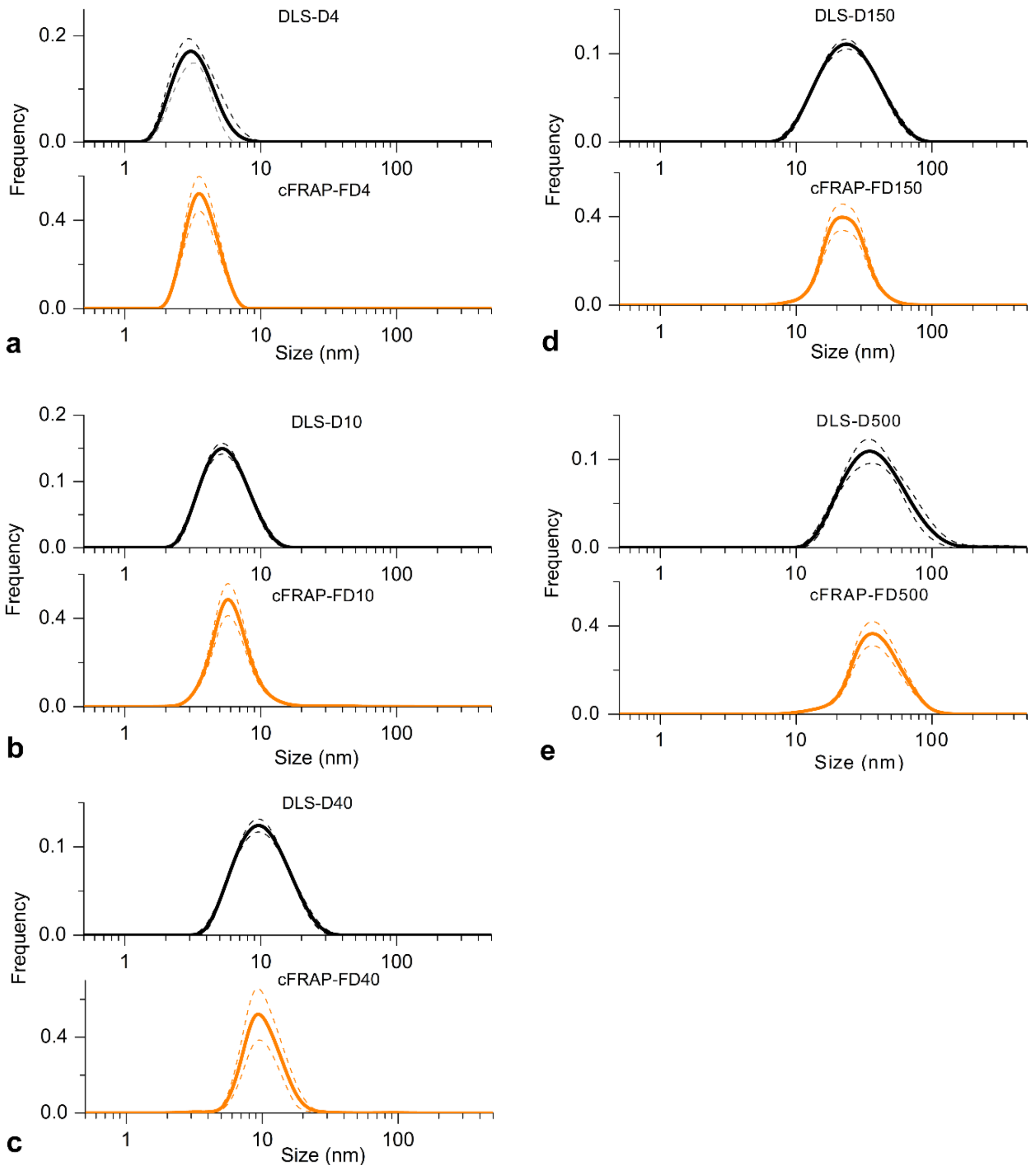

Supplementary Figure 8. Experimental validation of cFRAP versus DLS as a standard sizing technique of nanomaterial dispersions. Measurements are performed on five types of dextrans each with a different nominal MW: (**a**) 4 kD, (**b**)10 kD, (**c**) 40 kD, (**d**) 150 kD and (**e**) 500 kD. For cFRAP the dextrans were labelled with FITC (FD), while they were unlabelled (D) for DLS. The orange lines correspond to the cFRAP measurements, and the black lines correspond to DLS results. The dashed lines indicate the standard deviation (n = 10). Note that the same concentration of 0.5 mg ml^{-1} was used for cFRAP and DLS measurements.

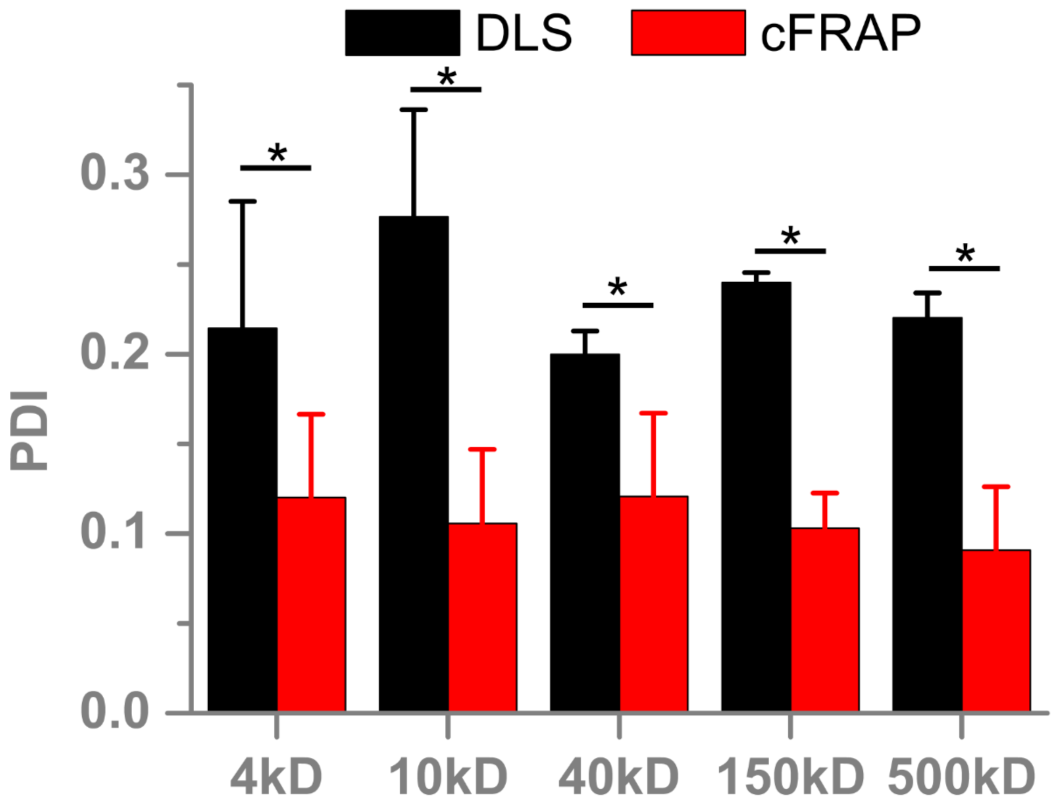

Supplementary Figure 9. Comparison of the apparent size polydispersity measured by cFRAP compared to DLS. As DLS essentially only uses time information of the diffusion process, its precision is significantly less as compared to cFRAP which also takes spatial information into account. (n=10, one-way ANOVA analysis, *P<0.05).

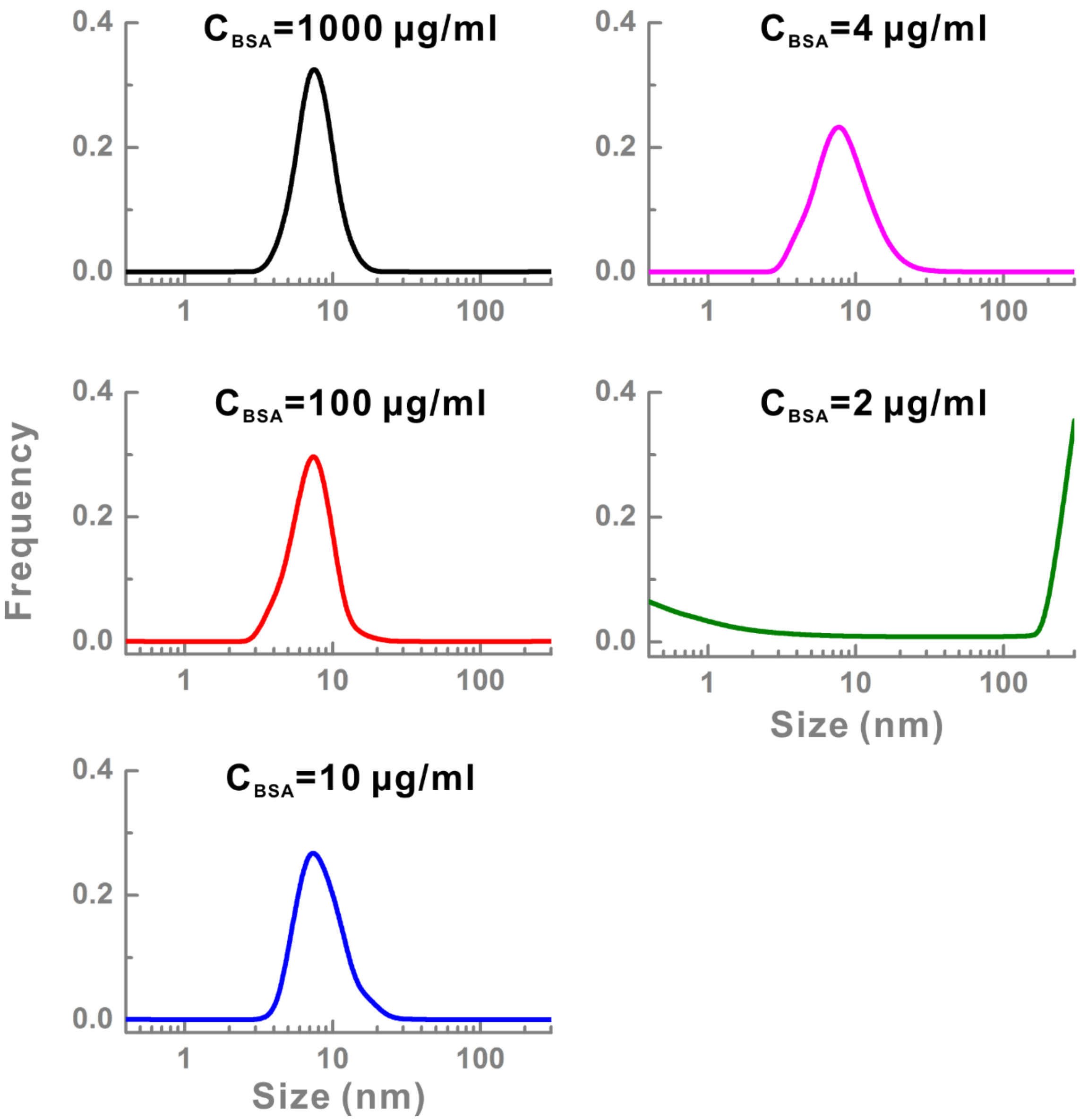

Supplementary Figure 10. Determination of the lower concentration limit for meaningful cFRAP analysis of labelled BSA. cFRAP was performed on solutions in PBS with decreasing BSA concentration from 1000 μg ml^{-1} (~15 μM) to 2 μg ml^{-1} (~30 nM). cFRAP analysis was possible down to a concentration of 4 μg ml^{-1} (60 nM).

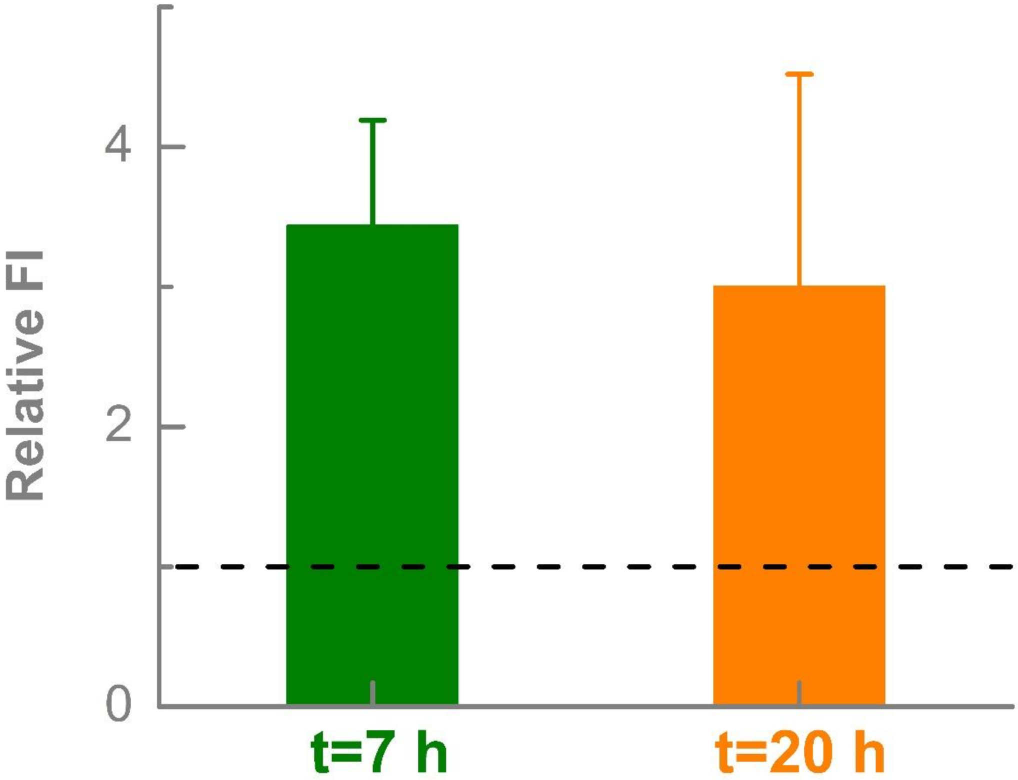

Supplementary Figure 11. The total fluorescence of FDs in blood after oral gavage in mice with septic shock was measured by fluorimetry (λ_{ex}=488 nm and λ_{em}=520 nm). The values (n = 3) are expressed relative to the fluorescence in control mice (indicated by black dashed line), which were injected intraperitoneally with PBS only. Blood samples were collected at t = 7 h (green bar) and t=20 h (orange bar).

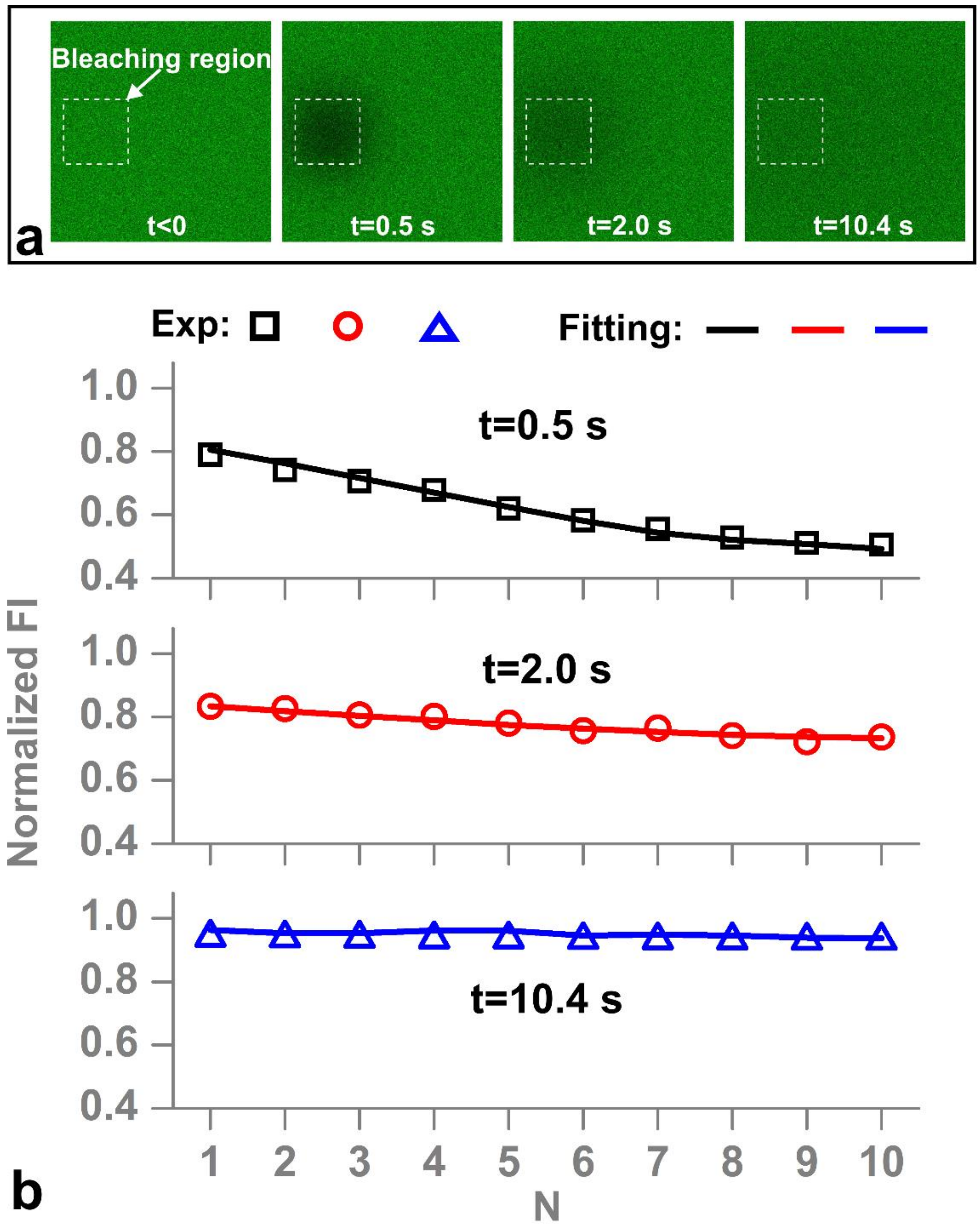

Supplementary Figure 12. Example of a typical cFRAP experiment performed in blood. (**a**) The blood sample was collected at 7 h after LPS injection of a mixture of FDs covering a broad range of sizes. Confocal time-lapse series showing images before (t<0) and after bleaching (t = 0.5, 2.0 and 10.4 s). (**b**) Examples of spatial recovery curves at different time points are shown. N is the ring number as explained in **Supplementary Figure 1**. The symbols denote the experimental data and the solid lines indicate the best fit solution of the cFRAP model to the data.

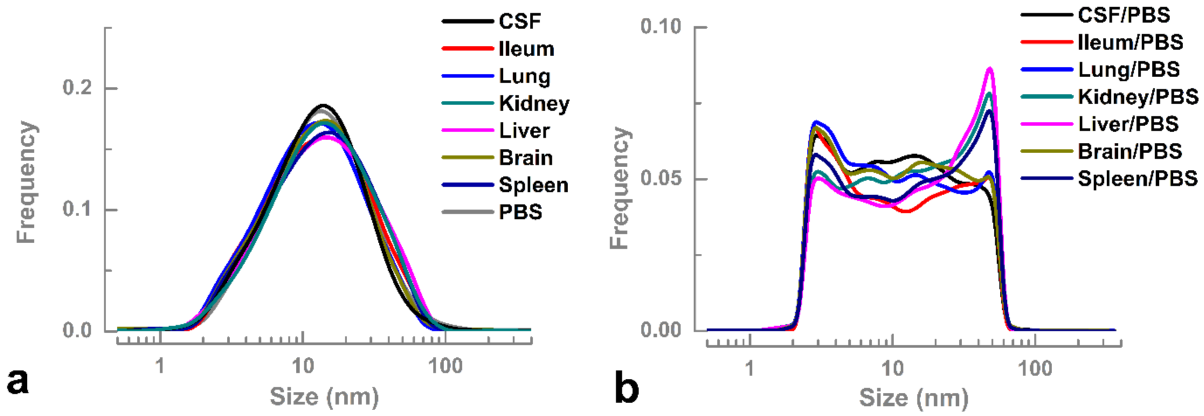

Supplementary Figure 13. To assess the permeability of the vascular barrier, a mixture of FITC-dextrans was prepared spanning a wide range of sizes from ~2 to ~80 nm. (**a**) The FD mixture was measured by cFRAP in PBS, CSF and extracted organs fluids. (**b**) Normalization of the size distributions in CSF or organ fluids to the size distribution in PBS buffer corrects for differences in concentration and fluorescence brightness between the various probes in the mixture. All of above data are the average of 10 measurements.

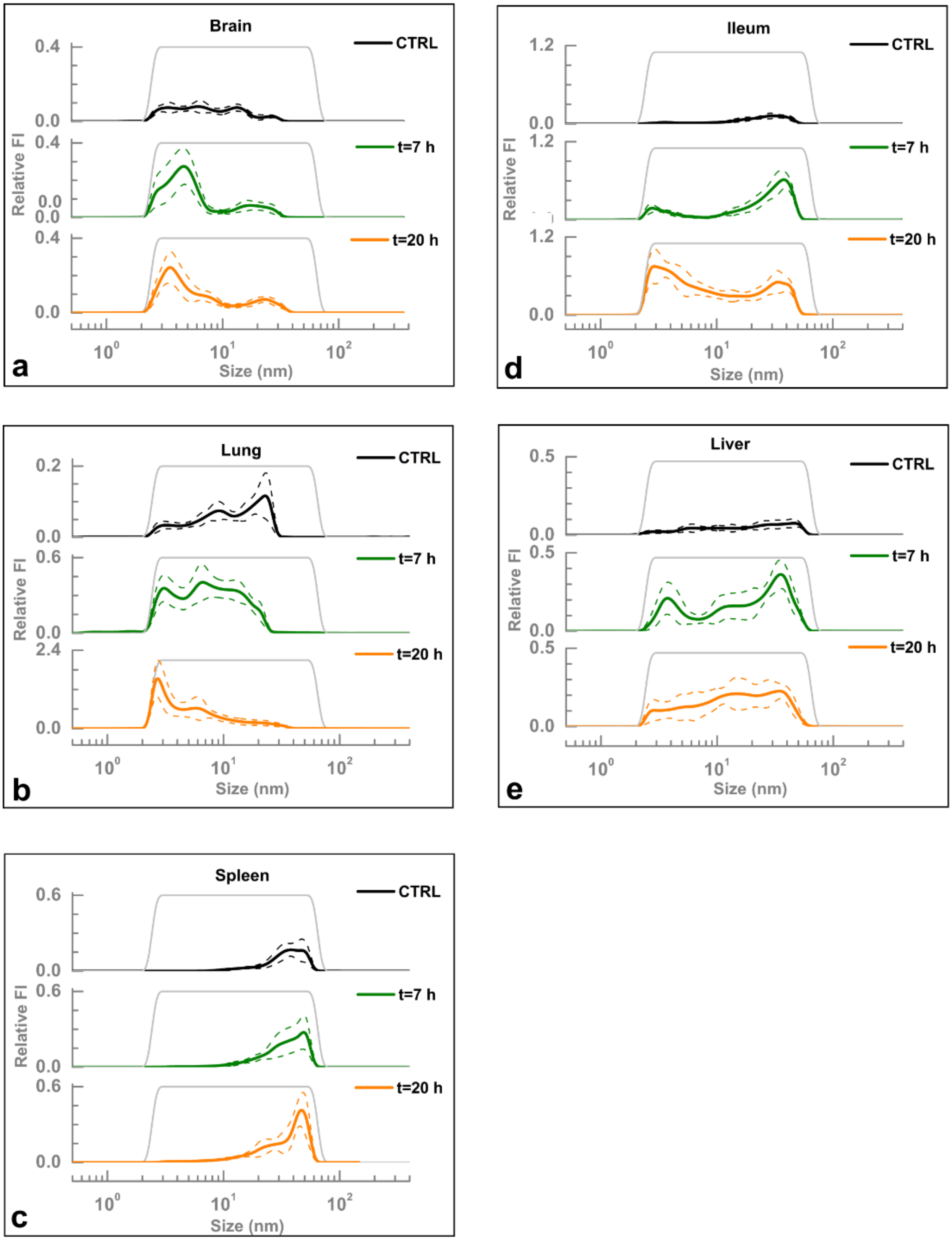

Supplementary Figure 14. See next page for caption.

Supplementary Figure 14. Assessing the vascular permeability in mice by measuring the range of probes that can permeate through the vascular endothelium in mice. cFRAP-sizing measurements were performed on (**a**) brain-, (**b**) lung-, (**c**) spleen-, (**d**) ileum- and (**e**) liver-fluids. Control mice were injected with PBS (instead of LPS) to determine the leakage in healthy mice as a reference (black lines). The data shown are the average from 3 mice, and for each mice 10 cFRAP-sizing measurements were performed. The solid line is the average of all these results and the dashed lines indicate the corresponding standard deviation. Note that direct comparison of the Relative FI values is only valid for the different time points of the same organ. Comparison of Relative FI values should not be made between organs. We could see brain an increase in the permeation of small molecules < 10 nm for LPS treated mice. The same is true for the lungs although they also contained a small fraction of large FDs (> 20 nm) after 20 h. In the spleen there was only an increase in the permeation of large molecules (> 20 nm) while in the liver probes were found over the entire tested range (~2 - ~80 nm). The Ileum, finally, exhibits a bimodal distribution of probes in LPS treated mice with a marked increase in the permeation of small molecules (~2 - ~10 nm). These data show that the cFRAP method enables detailed analysis of vascular permeability in all tested organs.

Supplementary Table 1. The overall fluorescence intensity in CSF and organ fluids as measured by fluorimetry. Samples were collected at time points of t=7 h and t=20 h after intraperitoneal injection of PBS (control) or LPS (septic choc) in mice. The mixture of FDs was IV injected 1h before sample collection. The fluorescence intensity values are shown as absolute fluorescence intensity (FI, arbitrary units) and relative to the fluorescence in control mice, which were injected with PBS only.

CSF or organ	PBS Injection (CTRL)	LPS Injection (t=7 h)		LPS Injection (t=20 h)	
	Absolute FI (mean±SD,×10^3)	Absolute FI (mean±SD,×10^3)	Relative FI (mean±SD)	Absolute FI (mean±SD,×10^3)	Relative FI (mean±SD)
CSF	0.07±0.05	0.35±0.38	5.1±7.2	0.45±0.06	6.6±0.9
Kidney	3.0±1.0	7.0±4.2	2.3±1.4	15.3±6.5	5.2±2.2
Brain	7.3±0.01	13.7±6.8	1.9±1.0	12.6±5.2	1.7±0.7
Lung	0.4±0.04	2.0±1.6	4.9±3.5	3.6±1.5	8.8±3.4
Spleen	2.2±0.02	34.5±11.7	1.6±0.5	42.6±16.8	1.9±0.8
Ileum	17.6±1.4	87.9±4.2	5.0±2.4	189.3±60.5	10.8±3.4
Liver	4.4±0.2	16.8±7.4	3.8±1.6	19.9±8.1	4.3±1.8

Supplementary Note 1: Pixel-based fitting vs. ring-averaged method

The rFRAP (rectangle FRAP) model presented here provides a complete description of the recovery phase in time and space after photobleaching. Therefore, fitting of the model can be done to all pixel values in a confocal time-lapse recording of the fluorescence recovery. Direct fitting to all pixel values in the ROI (120 by 120 pixels), however, results in very long calculation times. In order to speed up the fitting procedure, we evaluated a slightly modified approach where the spatial information (pixel values within a particular confocal image) is averaged over rectangular ring areas as illustrated in **Supplementary Fig. 1**. The ROI is divided into *n* rings according to $\Delta L=L/2n$, where ΔL is the spacing between each ring and L is the length of the ROI. We hypothesize that the same accuracy can be obtained as compared to fitting to all individual pixels on condition that the spatial increment of rectangular ring areas is sufficiently small. To evaluate this, we simulated recovery images of a single component system with a diffusion coefficient of 10 $\mu m^2 s^{-1}$. As the results show in **Supplementary Fig. 2**, the ring-averaged method has the same precision as pixel based fitting when the number of rings is larger than 10. Increasing the number of rings more did not result in more precision. Most importantly, the calculation time is reduced by 3 orders of magnitude when using the ring method (*n*=10) versus pixel-based fitting. An interesting special case is when a single ring is used, which corresponds to the most common way to analyse FRAP data where only the time-course of the average intensity in the bleach region is considered. As shown in **Supplementary Fig. 2** (NC=1, where NC is the number of divided rectangle ring), while the apparent D-distribution is still centred at the expected D-value of 10 $\mu m^2 s^{-1}$, it is much broader as compared to our new method where also the spatial information is included. This nicely demonstrates that cFRAP offers much better precision and resolution to analyse polydisperse samples as before.

Supplementary Note 2: Discretization of D-space

The cFRAP approach requires discretization of the D-space. Obviously, a finer discretization is expected to give a better description of the distribution, but comes at the expense of longer calculation times. Therefore, we evaluated the influence of the number of D-components (*ND*) on the fitting results. Recovery images

were simulated for a single component system with D = 10 μm^2 s^{-1}. The D-space was logarithmically discretized in *ND* components over 3 orders of magnitude. The results in **Supplementary Fig. 3** show that increasing *ND* from 20 to 30 slightly changed the resulting distribution, while the calculation time increases more than two fold. Based on these results, we decided to use *ND* of 20 or 30 for further experiments.

Supplementary Note 3: **Optimization of the total measurement time and the time interval between recovery images**

After photobleaching, time-lapse images are recorded of the recovery phase. The question then arises what is the most optimal time between subsequent images (Δt)? On the one hand it should be sufficiently short so as to capture all diffusion dynamics, while on the other hand one wants to limit total measurement recording time (T) by limiting total number of images to minimize (unwanted) photobleaching during imaging. To investigate what are suitable values for Δt and T, recovery images were simulated of a single component system with a diffusion coefficient of 10 μm^2 s^{-1}. To allow to draw general conclusions, Δt and T will be expressed relative to the characteristic recovery time, defined as $\tau = (l/2)^2/4D$, where l is the length of the shortest side of the bleach rectangle and D is the diffusion coefficient. As shown in **Supplementary Fig. 4a**, according to the single component system there is no improvement as soon as $T \geq \tau$. Looking at the result in **Supplementary Fig. 4b**, we conclude that Δt should be smaller or equal to 0.5τ. To study the influence of these experimental parameters in more detail for a complex polydisperse systems, we also simulated recovery images of a two-component system consisting 50% of D_1=1.0 μm^2 s^{-1} and 50% of D_2=10 μm^2 s^{-1}. Here, we defined τ_{slow} and τ_{fast} as characteristic recovery time of the slow and fast component, respectively The results are shown in **Supplementary Fig. 4c, d** for different Δt and T. Not unexpectedly the slow component cannot be accurately determined anymore when T becomes too short. Just as for the single component system we conclude that $T \geq \tau_{slow}$. Also regarding Δt we come to the same conclusion as from the single component system that $\Delta t \leq 0.5\tau_{fast}$.

Taken together, from these simulations we conclude that Δt should be at least 50% of the characteristic diffusion time of the fastest component, while the total measurement time should be at least equal to the characteristic diffusion time of the slowest component. For a fixed Δt this actually leads to over-sampling of the slower components so that more images are recorded than actually needed for the analysis, potentially leading to substantial photobleaching during imaging of the recovery phase. Therefore, we recommend the use of a linearly increasing of Δt with ~20% per image or, as in our case, a doubling of the time step per every 5 frames as shown in **Supplementary Fig. 4e, f**, until the selected total imaging time *T* is reached.

Supplementary Note 4: Discriminating and quantifying subpopulations with different diffusion coefficients

Having optimized experimental and analysis parameters, it is of interest to determine the resolution of cFRAP with which it can distinguish between subpopulations with different diffusion coefficients. Recovery images were simulated for a two-component system with different ratios of the diffusion coefficients ($R_D = D_2/D_1$). Both components are present in the same concentration (50/50%). The results are shown in **Supplementary Fig. 5a** for R_D=5, 3 and 2. For R_D=5 both components are found in the expected 50/50% ratio. Indeed, the area under the curve is 49.9% for the slow component and 50.1% for the fast component. Also at a ratio of R_D=3 both components are still well resolved, but at a ratio of 2 both populations could no longer be discriminated. Still this is an excellent result as compared to the standard approach of only taking the time-progression of the average intensity into account. This corresponds to $n = 1$ ring with our method, and in that case a ratio R_D at 8 is needed to discriminate both components as shown in **Supplementary Fig. 5b**.

Supplementary Note 5: Analysis of continuous *D* distributions

For parameter optimization and basic evaluation of the method until now we used single or double component systems. However, the cFRAP method was developed with the aim to analyze continuous distributions of diffusion coefficients. Therefore, we went on to simulate recovery images for a polydisperse

sample with a continuous distribution of diffusion coefficients according to a lognormal distribution with location parameter μ = 1.4 µm^2 s^{-1}and varying scale parameter σ from 0.1 to 2 to simulate an increasingly polydisperse system (black bars in **Supplementary Fig. 6a-c**). The orange curves are the result from cFRAP analysis and nicely corresponds to the expected distributions. Going one step further we also simulated a double lognormal distribution with μ_1=0.9 µm^2 s^{-1}, σ_1 =1.5 and μ_2=8.7 µm^2 s^{-1} σ_2=1.5. As shown in **Supplementary Fig. 6d**, again cFRAP is very well capable of retrieving the expected distribution. This demonstrates that cFRAP is very well suited for the intended task of analyzing complex polydisperse systems.
